# Enabling FAIR data in Earth and environmental science with community-centric (meta)data reporting formats

**DOI:** 10.1038/s41597-022-01606-w

**Published:** 2022-11-14

**Authors:** Robert Crystal-Ornelas, Charuleka Varadharajan, Dylan O’Ryan, Kathleen Beilsmith, Benjamin Bond-Lamberty, Kristin Boye, Madison Burrus, Shreyas Cholia, Danielle S. Christianson, Michael Crow, Joan Damerow, Kim S. Ely, Amy E. Goldman, Susan L. Heinz, Valerie C. Hendrix, Zarine Kakalia, Kayla Mathes, Fianna O’Brien, Stephanie C. Pennington, Emily Robles, Alistair Rogers, Maegen Simmonds, Terri Velliquette, Pamela Weisenhorn, Jessica Nicole Welch, Karen Whitenack, Deborah A. Agarwal

**Affiliations:** 1 Earth and Environmental Sciences Area, Lawrence Berkeley National Laboratory, Berkeley, CA 94720 USA; 2 Environmental Studies Department, California State University, Sacramento, 6000 Jed Smith Dr, Sacramento, CA 95819 USA; 3 Argonne National Laboratory, Lemont, IL 60439 USA; 4 Pacific Northwest National Laboratory, Joint Global Change Research Institute at the University of Maryland–College Park, College Park, MD 20740 USA; 5 Environmental Geochemistry Group, SLAC National Accelerator Laboratory, 2575 Sand Hill Road, Menlo Park, CA 94025 USA; 6 Scientific Data Division, Lawrence Berkeley National Laboratory, Berkeley, CA 94720 USA; 7 Environmental Sciences Division, Oak Ridge National Laboratory, Oak Ridge, TN 37830 USA; 8 Environmental and Climate Sciences Department, Brookhaven National Laboratory, Upton, NY 11973 USA; 9 Pacific Northwest National Laboratory, Richland, WA 99354 USA; 10 Integrated Life Sciences, Virginia Commonwealth University, Richmond, VA 23284 USA; 11 Present Address: Pivot Bio, 2910 Seventh Street, Berkeley, CA 94710 USA; 12 Present Address: Github, San Francisco, CA 94107 USA

**Keywords:** Environmental social sciences, Databases, Environmental sciences

## Abstract

Research can be more transparent and collaborative by using Findable, Accessible, Interoperable, and Reusable (FAIR) principles to publish Earth and environmental science data. Reporting formats—instructions, templates, and tools for consistently formatting data within a discipline—can help make data more accessible and reusable. However, the immense diversity of data types across Earth science disciplines makes development and adoption challenging. Here, we describe 11 community reporting formats for a diverse set of Earth science (meta)data including cross-domain metadata (dataset metadata, location metadata, sample metadata), file-formatting guidelines (file-level metadata, CSV files, terrestrial model data archiving), and domain-specific reporting formats for some biological, geochemical, and hydrological data (amplicon abundance tables, leaf-level gas exchange, soil respiration, water and sediment chemistry, sensor-based hydrologic measurements). More broadly, we provide guidelines that communities can use to create new (meta)data formats that integrate with their scientific workflows. Such reporting formats have the potential to accelerate scientific discovery and predictions by making it easier for data contributors to provide (meta)data that are more interoperable and reusable.

## Introduction

Making Earth and environmental science data Findable, Accessible, Interoperable, and Reusable (FAIR)^1,2^ contributes to research that is more transparent and reproducible^3^. Search engines and data repositories^2,4,5^ have enabled advances in data preservation, findability, and accessibility. However, data interoperability and reuse remain major challenges in part due to the diversity of Earth science data, and because researchers may lack time and funding for data management or awareness of tools and resources to make data more reusable^5,6^. This results in barriers to scientific research and knowledge generation; for example, synthesis of data across different sources can be extremely time-consuming when data and metadata are not standardized in a common, well-defined format.

Standards for data and metadata, hereafter referred to as (meta)data standards, have been proposed as important elements to make Earth and environmental science data easier to find, understand and reuse^7–10^. Formal (meta)data standards are typically accredited by large governing bodies and emphasize making data broadly reusable^11^. For example, the International Organization for Standardization (ISO) 8601 standard provides guidelines for formatting date and timestamps and has been adopted in a wide range of research and business sectors^12^. The Open Geospatial Consortium’s Sensor Observation Service standard^13^ outlines standardized ways of pulling sensor data from web interfaces. Such accredited standards are extraordinarily useful, but are available only for a few environmental data types and can take over a decade to build governing processes and consensus.

In contrast, reporting formats are community efforts aimed at harmonizing diverse environmental data types without the oversight of the governing protocols or working groups that maintain vocabularies and extensive documentation. There are reporting formats for different research domains and data types including water quality^14^ and meteorological data^15^. Reporting formats are typically more focused within scientific domains—for example, marine observations^16^ or solid earth geoscience^17^. Reporting formats can enable efficient collection and harmonization of information needed to understand and reuse specific types of data within a research community. For example, the use of FLUXNET’s half-hourly flux and meteorological reporting format^18^ enables both access and reuse of consistently formatted carbon, water, and energy flux data from thousands of sampling locations across the world. However, reporting formats do not exist for most environmental data types, and even if they do, complexity and lack of resources can limit their adoption^9^.

There are many scientific benefits when research communities adopt reporting formats, ranging from organizing data collection in the field or lab to more efficient data reuse in synthesis and modeling efforts. Reporting formats can facilitate data sharing within a group, provide guidelines for consistent data collection, enable streamlined scientific workflows, and enable long-term preservation of knowledge that may not be typically stored or reported with the data^19,20^. Moreover, research disciplines are beginning to operationalize and implement practices^21,22^ to achieve the original FAIR guiding principles^21,22^. Reporting formats developed by the research communities for which they are intended are seen as a critical step toward achieving greater data interoperability and reuse^22^.

A variety of multidisciplinary data are generated in research sponsored by the U.S. Department of Energy (DOE) and stored in the Environmental Systems Science Data Infrastructure for a Virtual Ecosystem (ESS-DIVE) data repository^4,23^. Integration and analysis of diverse data types such as hydrological, geological, ecological, biological, and climatological data is an essential element of complex environmental systems science (ESS) research. However, such interdisciplinary data integration presents unique challenges, such as inconsistent use of terms, formats, and metadata across disciplines^24^. In this manuscript, we describe and harmonize 11 diverse and complementary (meta)data reporting formats that our interdisciplinary team developed for commonly used data types in ESS research to enable their archival following FAIR principles in general purpose repositories such as ESS-DIVE. These include guidelines to format and describe general research elements (e.g., general file metadata, tabular data, physical samples, model data), as well as guidelines developed for more specific data types relevant to interdisciplinary research (e.g., biogeochemical samples, soil respiration, leaf-level gas exchange). As part of this process, we adopted or used components of existing reporting formats or standards to the greatest extent possible, and also developed new reporting formats for some data types. These can be used individually or collectively in scientific workflows, and many of the formats are widely applicable for environmental research. Moreover, the process we used for developing the formats—including our approach to obtain community consensus, mirror documentation across several web platforms, and track community feedback—can be used by other research communities to develop reporting formats for their own purposes.

## Results

Our community-centric approach to developing reporting formats had four key outcomes that are broadly important to making scientific data more reusable. First, the teams reviewed a total of 112 pre-existing data standards and other data resources (data repositories, data systems, datasets, projects) to create (meta)data crosswalks (Supplementary Files 1–20). Such crosswalks provide a tabular map of existing resources related to each data type, allowing the teams to identify gaps in existing standards, and determine which variables, terms, and metadata were essential to harmonize and incorporate into their reporting formats. At the onset of the review process, ESS-DIVE recommended adopting existing standards to the extent possible. However, we found that for all 11 data types, none entirely met ESS research community needs, and this necessitated development of all 11 reporting formats.

**Fig. 1:**
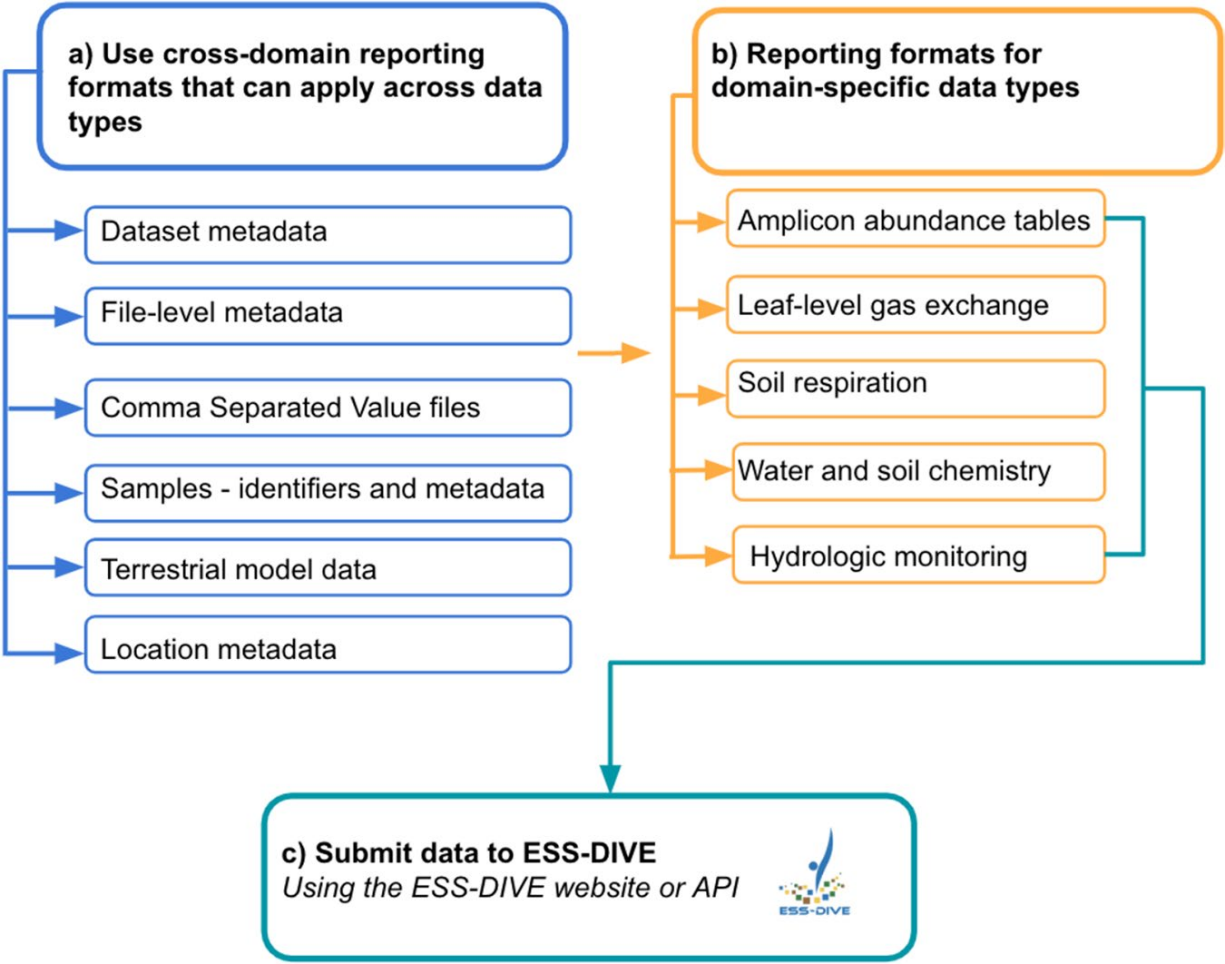
Workflow to help determine which (meta)data reporting formats apply to datasets. The set of 11 ESS-DIVE (meta)data formats are either (**a**) cross-domain guidelines that can be applied to many data types or (**b**) are data type-specific. For those archiving data with ESS-DIVE, researchers can upload data through the ESS-DIVE web user interface^155^ or programmatically through an API.

Second, we created 11 reporting formats (Supplementary Table 1) that encompass a range of complex and diverse ESS (meta)data fields that can be used when researchers upload data to ESS-DIVE. Six of the reporting formats created by our community of scientists are cross-domain reporting formats (Fig. 1a), which apply broadly to data across different scientific disciplines. These reporting formats were developed to help researchers more consistently format their (meta)data for interdisciplinary science applications and include basic dataset metadata for citation and findability^25^, file-level metadata^26^, guidelines for formatting comma separated value (CSV) files^27^, sample metadata^28^, terrestrial model data archiving guidelines^29^, and research locations metadata^30^. The remaining five reporting formats apply to different domain data types (Fig. 1b) and include microbial amplicon abundance tables^31^, leaf-level gas exchange^32^, soil respiration^33^, sample-based water and soil chemistry measurements^34^, and water level and sonde-based hydrologic measurements^35^. All reporting formats have a minimal set of required metadata fields necessary for programmatic data parsing and optional fields that provide detailed spatial/temporal context about the sample useful to downstream scientific analyses. Throughout development, we aimed to strike a balance between pragmatism for the scientists reporting data and machine-actionability that is emblematic of FAIR data. A comparison between FAIR guiding principles and our reporting formats (Supplementary Table 2) highlights how a community-centric effort like ours can move data archiving towards achieving many FAIR data principles (though see discussion for limitations).

Together, these 11 reporting formats are part of a flexible, modular, and integrated framework (Fig. 1) that can accommodate new reporting formats in the future, and enable their findability and accessibility individually or collectively. As part of the framework development, all teams created templates with harmonized terms and formats to be internally consistent as much as possible. For example, dates are always reported in YYYY-MM-DD format. Whenever reporting formats include spatial data, the variables are harmonized as “latitude” and “longitude” and reported in decimal degrees with common bounds (−90 to 90 and −180 to 180, respectively). All formats that require CSV files adopted as many recommendations from the CSV reporting format as possible. Data collected using the water and soil chemistry, and amplicon reporting formats have an option to report a persistent identifier for associated samples [International Generic Sample Number (IGSN)], to enable effective tracking across online data systems, as outlined in the Sample ID reporting format.

The third outcome is related to how we shared and archived all reporting formats in three ways, each with a distinct use. First, all reporting formats are published as datasets in the ESS-DIVE repository, which enables direct, public download and citation upon use. Second, each reporting format is hosted on the version control platform GitHub, which enables ongoing edits and versioning while also allowing users to provide feedback^36^. Third, the most up-to-date reporting format content from GitHub is rendered as a project website through the service GitBook^37^. We mirrored the reporting format instructions and templates across several web platforms to ensure the documentation is available in a variety of digital formats to serve the needs of various user groups and stakeholders. GitHub is likely a more familiar platform and user interface for software engineers and informatics specialists, for example, while GitBook websites may be preferred by Earth science researchers.

Lastly, we formulated guidelines (Box 1) for research communities that want to replicate our model of community-centric (meta)data reporting format development. We encourage (1) reviewing existing standards, (2) developing a crosswalk of terms across relevant standards or ontologies of interest, (3) iteratively developing templates and documentation with feedback from prospective users, (4) assembling a minimum set of (meta)data required for reuse, and (5) hosting finalized documentation on platforms that can be publicly accessed and updated easily.

Box 1 Guidelines for research communities to self-organize and create, document, and share (meta)data reporting formats when formats do not already exist or do not fit scientists’ needs. 1. **Research existing (meta)data standards and other data resources** across agencies and organizations both within the US and internationally.

**Fig. 2:**
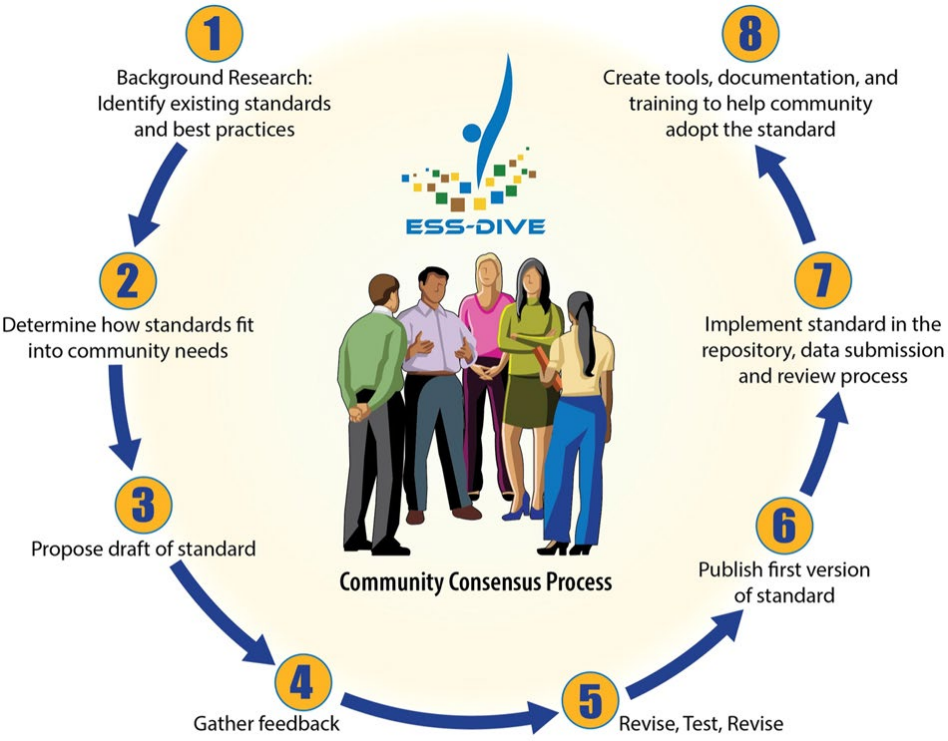
Each of the 11 ESS-DIVE (meta)data reporting formats were developed in cross-functional teams that often involved domain scientists, software engineers, and informatics specialists.

2. Create a **(meta)data crosswalk** (Supplementary Files 1–10) to define how other standards and data resources translate to the proposed reporting format.

3. Work with the scientific community to **iteratively develop and obtain feedback** (see Fig. 2) on (meta)data reporting format.

4. Develop **documentation** (instructions, templates, variables, descriptions, units, metadata) to support the format. Consider appropriate **file formats** for any templates.

5. Archive finalized version of the reporting format in a **long-term data repository** as well as a version control platform (e.g., GitHub^37^).

## Discussion

Many scientific journals and funders require data deposition in long-term repositories. However, in many cases, data are submitted to repositories in bespoke formats with little (meta)data standardization^5^. Community-led (meta)data reporting formats like the set described in this paper can enable archived data to be more reusable and interoperable^21,22^. Our scientist-centric approach to creating the formats helped to determine workflows that are most useful and practical for researchers to adopt. Here we discuss important aspects that need to be considered in development and use of such reporting formats.

Reporting formats can help researchers organize and synthesize their own (meta)data for their research purposes. It can be challenging for small teams, or even individuals to keep track of data collected over multi-year field campaigns or laboratory experiments^19,20^. Early adoption of a consistent way of compiling data can help individuals or research teams avoid ad hoc data collection practices and also help researchers efficiently integrate their data, particularly when multiple analyses or teams are involved.

Moreover, community reporting formats can lead to greater data accessibility and reuse. For example, researchers in the Ameriflux network^38^ organize flux data in the Flux Processing (FP-in) reporting format^18^. When participants in the network agree to provide their flux data in this format^39^, benefits include: 1) access to data services such as automated QA/QC of datasets and value-added ONEFlux data processing^40^, 2) Digital Object Identifier assignment which helps to track dataset citation and reuse, and 3) potential to increase findability of their data. Similarly, when contributors upload datasets on ESS-DIVE, they are offered automated metadata quality assessments, and published data are assigned DOIs and made searchable across the DataONE network. In another example, the Watershed Function Scientific Focus Area project^41^ adopted ESS-DIVE’s water and soil chemistry reporting format as an initial step towards establishing a field data workflow in a community observatory where diverse hydrological, geochemical, geophysical, ecological, and remote sensing datasets are collected^42^. The use of the reporting format will make it possible for researchers to synthesize data on chemical concentrations both within and across field locations.

Application of the reporting formats also allows for the use of tools and services that enhance data curation, findability and reuse. As an example, some of the fields in ESS-DIVE’s dataset metadata reporting format^25^ allow programmatic metadata quality validation, which checks for field presence, format, and length. Because these metadata can be mapped to a variety of machine-readable metadata formats including JSON-LD and the U.S. Department of Energy’s Office of Scientific and Technical Information (OSTI) reporting formats^43^. This further enabled transforming and disseminating ESS-DIVE datasets across other platforms such as Google Dataset Search, DataONE, OSTI and DataCite.

The development of these reporting formats was driven by the scientific need for practical tools for data management, while improving the potential for data reuse achieving many of the FAIR guiding principles (Supplementary Table 2). We made several pragmatic choices to ensure that the reporting formats would have a low barrier to adoption by time-limited researchers. This included investigating whether using pre-existing reporting formats “off the shelf” would meet project and researcher’s scientific needs and workflows. Although it is desirable to use existing formats whenever possible, we found that there were many circumstances when they do not directly apply to a scientific community’s research (meta)data needs. For example, although the Water Quality Exchange format^14^ is used within the United States to report water quality monitoring data by local, state, and federal agencies, the format was not entirely suitable for ESS-DIVE’s purposes. Some of the concerns raised by the community included: 1) the structure of the data and metadata templates that are used for regulatory reporting were considered to be cumbersome and inefficient for scientific use (e.g., containing redundant elements of sampling and analytical methodology along with the data) and 2) the required vocabularies (as specified in the template dictionary) were found to be difficult to use because they included several terms that were unnecessary, while missing terms for specific analytes of interest to the community.

To address these concerns, we developed the ESS-DIVE reporting format for sample-based water and soil chemistry^34^ that is more suitable for files typically generated in scientific laboratories. It borrows elements from the WQX standard, but provides flexibility in format and terminology, while capturing sufficient metadata and vocabularies to enable data exploration and reuse including the ability to use scripts to compare and combine different datasets^34^. In this way, the water and soil chemistry reporting format achieves some component of FAIR guiding principle “I2” that suggests using ontologies, while still being responsive to a research community that desired flexibility in research terminology (Supplementary Table 2).

Similarly, when creating the sample ID metadata reporting format, we decided to extend the existing IGSN sample identifier template and guidelines in ESS-DIVE’s Sample ID reporting format to meet researchers’ need to link interdisciplinary environmental and biological samples, and to minimize effort in providing information for sample collections^44^. In this case, incorporating IGSNs ensures that researchers using this format achieve FAIR principle “F3” and have globally unique identifiers for their data products, which facilitates tracking associated sample data across multiple online data systems. In an effort to be pragmatic, we decided to lower the threshold for adoption of the sample ID reporting format (and nearly all others; Supplementary Table 2) by compromising on elements that would achieve FAIR principle “I3” related to machine readable knowledge representation. All reporting formats encourage users to define variables in a data dictionary. Though this may not be fully machine readable according to the FAIR principles^21^, this method of defining variables is a key step toward reusable and machine actionable data. The feedback gathered when creating our Sample ID reporting format was then provided to the broader IGSN community to help improve the IGSN metadata template for interdisciplinary science^45,46^.

Through the process, we learned that many (meta)data standards are not accessible to a typical researcher and require a significant learning curve to become fluent in the informatics terminology used by established data standards. For example, the Open Geospatial Consortium’s data standard for environmental sensors^13^ is a detailed schema described over 100 pages, which is challenging for a typical scientific researcher to understand and implement. Hence, we had to make several pragmatic choices to ensure that the reporting formats would be amenable to adoption by time-limited researchers. Once choice involved replacing terms in existing standards with words that were more intuitive to scientists. For example, whilst there was no reporting format for leaf-level gas exchange data, a crosswalk of the instrument output from a relatively small number of instrument manufacturers quickly identified a common terminology that already had broad acceptance and use by the scientific community (Supplementary File 7). By using crosswalks (Supplementary Files 1–10) our teams were able to map ESS-DIVE’s reporting formats to many existing (meta)data standards and other data resources, and, in the future, will allow building tools that enable interoperability with different systems. We also simplified the reporting format templates and instructions to the greatest extent possible by specifying a few required fields and several more optional fields to provide additional details.

Our model and guidelines of supporting and empowering the scientific community to develop (meta)data reporting formats that meet their needs can enable other communities to undertake these internal data standardization efforts that make their data even more useful beyond the purpose for which they were collected (Box 1). We acknowledge that other research infrastructures have made important strides toward data standardization within research communities though they can still take dozens of years to manifest^17^. We found value in including a broad range of stakeholders in the process, and included field personnel who make the measurements, instrument manufacturers, and scientists who use the data in models or synthesis activities^47^.

There are incentives that can help promote widespread adoption of these or other formats to justify the time investment required for individual researchers or teams into scientific workflows. First, involving data collectors and reusers at the core of the development process makes the resulting formats more pragmatic and scientifically useful. Importantly, the domain scientists involved in the reporting format development became community ambassadors and helped engage their use by fellow researchers through conference presentations and peer-reviewed papers^44,47–49^. Second, we expanded our user community by sharing information about the reporting formats through a series of webinars, documentation, tutorials, and personalized community outreach. These incentives have had some success, as evidenced by the datasets submitted to ESS-DIVE using one or more of the reporting formats within a few months after they were finalized (Table 1).

**Table 1:** Examples of datasets published on ESS-DIVE utilizing at least one of the 11 ESS-DIVE (meta)data reporting formats.

Dataset Title	Reporting Format(s) Used
FTICR, NPOC, TN, and Moisture of Variably Inundated Sediment across 48 North American Rivers^148^	Sample-based water and soil chemistry, Sample ID and metadata, Comma Separated Value files, and File-level metadata Reporting Formats
Kinetic and temperature sensitivity properties of soil exoenzymes through the soil profile down to one-meter depth at a temperate coniferous forest (Blodgett, CA)^149^	Sample ID and metadata, Comma Separated Value files, and File-level metadata Reporting Formats
Leaf Photosynthetic Parameters: Quantum Yield, Convexity, Respiration, Gross CO2 Assimilation Rate and Raw Gas Exchange Data, Utqiagvik (Barrow), Alaska, 2016^150^	Leaf-level gas exchange Reporting Format
Perceived Costs and Benefits of ICON Science and Foundational Documents associated with “Integrated, Coordinated, Open, and Networked (ICON) Science to Advance the Geosciences: Introduction and Synthesis of a Special Collection of Commentary Articles^151^	File-level metadata Reporting Format
FTICR-MS, Sensor, and Environmental Data from 5 Streams Impacted by the 2020 Holiday Farm Fire Associated with: “Spatiotemporal controls on the delivery of dissolved organic matter to streams following a wildfire^152^	Hydrologic Monitoring, Comma Separated Value files, and File-level metadata Reporting Formats
Fungal and bacterial growth variation due to drought and nitrogen addition experimental treatments^153^.	File-level metadata and Comma Separated Value files Reporting Formats
Chemistry data from soils and soil incubation experiments from the whole-soil warming experiment at Blodgett Forest, CA, 2018, from: “Metabolic capabilities mute positive response to direct and indirect impacts of warming throughout the soil profile”^154^	File-level metadata and Comma Separated Value files Reporting Formats

Each row includes the dataset title, citation, and the reporting format(s) used in the dataset.

We identify some future work that can potentially lower the barrier to adopting reporting formats, provide added benefits to those who use the formats, and make (meta)data FAIRer^10^. Currently, ESS-DIVE applies a set of manual checks to datasets uploaded to ESS-DIVE that follow the reporting format. However, development of automated formatting checkers^50^ would help users instantly validate their datasets against reporting format guidelines. Other types of software can also be built around the reporting formats. For example, software could be developed to automatically convert sensor or instrument-derived data into the units requested by a reporting format. As a starting point for this work, the file-level metadata reporting format already includes an open-source script^51^ that enables reading and parsing data files submitted in that format. The leaf-level gas exchange reporting format includes a detailed translation table matching the reporting format data variables with standard outputs from 10 commonly used, commercially produced instruments. This could provide the foundation for development of conversion software to automatically format data with the recommended variable names and units. ESS-DIVE is also planning a data integration and fusion component of the repository that will facilitate synthesizing and analyzing datasets that adhere to any of the 11 ESS-DIVE reporting formats. Enabling advanced queries within the files will require development of software and data parsers so that a great number of reporting formats achieve FAIR principle “F4” which calls for data to be fully searchable.

With more data being generated than ever, reusable data can have substantial societal, economic, and scientific impacts. But for Earth and environmental science data, which are complex and heterogeneous, achieving reusability will require concentrated effort at (meta)data standardization within research communities. Our work to develop 11 community (meta)data reporting formats is a critical step to making Earth and environmental science data more reusable because we emphasize human readability that is compatible with machine readability. We hope that our model of empowering research communities to self-organize and create their own (meta)data reporting formats will enable other communities to undertake these internal data standardization efforts that make their data even more useful beyond the purpose for which they were collected.

## Methods

Earth and environmental science data are complex, multi-scale, and span diverse research domains such as geology, hydrology, climate, ecology, and biology. At ESS-DIVE, we initiated a community-centric model that engaged domain scientists to develop formats for common Earth science data types. The objective was to create formatting guidelines and templates that would gather the minimum but sufficient metadata or data necessary for data interpretation and reuse.

### Reviewing existing standards and feedback on drafts

Each team conducted a review of existing standards (Supplementary Table 1), involving both literature searches and exploring resources from informatics groups (e.g., Research Data Alliance and Earth Science Information Partners) or agencies working with similar data, to identify whether any existing data standards or conventions could be used directly or to inform their reporting format. Based on this review, each team created tabular ‘crosswalks’ (Supplementary Files 1–10) to map related terminology from relevant standards. This process helped identify gaps in existing standards, and determine important elements that had to be present, and variations in terminology used across different standards that required harmonization. For example, some existing standards report date and time under the column name ‘datetime’ while another reports the same information, as ‘ValueDateTime’ (see example of a terminology crosswalk^35^). Here, we provide a brief narrative of methods for each reporting format with details on existing data standards and other data resources reviewed during reporting format development. For further details on the technical aspects of each reporting format, please refer to ESS-DIVE’s community space on GitHub^36^ or view the datasets for each reporting format submitted to ESS-DIVE (Supplementary Table 1).

### Obtaining community consensus

Each team created instructions and (meta)data templates for their reporting formats. The teams piloted the formats within their research groups and communities to ensure the templates were practical and useful for scientists who collect and reuse data (Fig. 2). In total, 247 individuals representing 128 institutions provided input at various stages of the reporting format development process. As the reporting format instructions and templates reached a final stage, they published the “ready-to-use” reporting formats in three locations each with distinct benefits for the end-users: GitHub^37^, GitBook, and the ESS-DIVE data repository to enable findability and long-term preservation.

### Cross-domain reporting formats

#### Dataset metadata

The goal for creating the dataset metadata reporting format was to ensure that any dataset submitted to ESS-DIVE would have complete and descriptive metadata to enable its findability and citation upon use. The ESS-DIVE team reviewed machine and human-readable metadata standards including the Ecological Metadata Language^52^ as well as JSON for Linking Data^53^. The ESS-DIVE metadata reporting format follows existing metadata standards as much as possible (e.g., ‘title’ in Ecological Metadata Language is also ‘title’ for ESS-DIVE’s metadata).

#### File-level metadata

The file-level metadata reporting format was developed for users to provide details about the individual files contained within a dataset. The review of existing standards^26^ included file-level metadata used across 6 organizations (e.g., USGS, NEON).

#### CSV file formatting guidelines

The CSV reporting format was developed to provide guidelines for more consistently formatting tabular data^27^. The intention was to make this a domain agnostic set of guidelines so that anyone who works with tabular data can use the format in their research to make tabular data more interoperable and machine-readable. The team reviewed existing standards and guidelines (Supplementary Table 1) including recommendations from the Environmental Data Initiative (e.g., do not mix data types in a column) and the ORNL DAAC (e.g., indicating missing numeric values with −9999).

#### Sample IDs and metadata

The ESS-DIVE Sample ID reporting format^28^ aligns as much as possible with extensive work on IGSN^54^ with the goal of standardizing sample collection metadata and more efficiently tracking physical samples sent to different collaborators, labs, data systems, etc. This work also reviewed 12 different standards and data resources to provide recommendations for improving interoperability of biological^8,55^ and environmental samples^14^.

#### Terrestrial model data archiving guidelines

The model data archiving reporting format^29^ was informed by input from the DOE’s land modeling community and other guidelines from the American Geophysical Union and National Science Foundation Earthcube communities. In developing the guidelines^49^, the goal was to help modelers make decisions about which components of their terrestrial models should be archived in a long-term data repository. The guidelines were developed with input on which model data were most useful to archive, how long they remained useful, and what scientific purpose they would serve.

#### Location metadata

The goal of developing the location metadata reporting format was to provide generalized guidelines for describing locations used in research. The review of existing standards included metadata templates from specific projects at some of the DOE’s National Labs to understand the different field sampling strategies of large interdisciplinary projects. The review also included known standards and guidelines for recording locations such as Climate and Forecast Conventions^56^, the Federal Geographic Data Committee’s Content Standard for Digital Geospatial Metadata^57^ and the Open Geospatial Consortium^58^.

#### Reporting formats for domain-specific data types

In addition to the set of 6 cross-domain reporting formats described above, we also developed 5 formats that are tailored to specific data types commonly used in the terrestrial and subsurface ecosystem research community. ESS-DIVE’s goal was to engage Earth and environmental scientists to determine practical reporting formats that data contributors are willing to use while at the same time ensuring a high potential for data reuse.

#### Amplicon abundance table metadata

The reporting format for amplicon abundance table metadata was developed to facilitate consistent reporting of microbiome sample data with the format of these tables following ESS-DIVE’s CSV file guidelines. Required data (e.g., representative sequences) were chosen to support comparisons of abundance tables across studies. The reporting format distinguishes between sequencing metadata and bioinformatic processing metadata for amplicon abundance tables. As much as possible, the team aligned recommendations for sequencing metadata with the existing standards developed by the Genomic Standards Consortium for minimum information about a marker gene sequence and minimum information about any (x) sequence^55^ (Supplementary File 6). In the absence of an existing standard for bioinformatic processing metadata, the reporting format contains a minimal set of fields to capture the data processing steps most relevant for comparing and combining amplicon counts across studies (Supplementary Table 1). The final set of sequencing and bioinformatic metadata fields selected were informed by a community of scientists involved with either the development of microbiome data pipelines or conducting microbiome studies in both field and lab settings.

#### Leaf-level gas exchange

The team working on this reporting format^32^ reviewed existing conventions used in plant trait databases, large data collections developed for synthesis papers, and the variable descriptions that are part of standard instrument outputs in order to determine the most suitable variable names to use to report leaf-level gas exchange data. Templates for formatting metadata about the methods and sample materials used in an experiment, as well as details on the instrumentation involved in collecting data were developed through an iterative process of input and review open to all interested stakeholders. The reporting format is designed to be flexible and modular, provides guidelines on the archive of raw and processed data, and seeks to capture experimental metadata needed to interpret and reuse these data types^47^.

#### Soil respiration

To create the soil respiration reporting format, this team reviewed and integrated recommendations from 9 existing guidelines and standards (Supplementary Table 1)^33^. The review captured an array of how different standards format their general metadata and data (e.g., formatting date and timestamps) and also accounted for a range of soil-atmosphere gas exchange data types (e.g., GHGs or radiocarbon)^48^.

#### Sample-based water and soil chemistry measurements

The goal in creating a reporting format for water-soil-sediment data was to harmonize chemical concentration data that span several measurement types. The review included 15 standards (Supplementary Table 1) for related environmental chemistry measurements including metadata elements from the EPA’s WQX^14^ as well as EarthChem^59^. Based on input from the potential ESS user community that included both data collectors, managers, and modelers, we developed a reporting format based on community input^34^.

#### Water level and sonde-based hydrologic monitoring

This reporting format harmonizes variables common to sonde-based hydrologic monitoring research including water level, temperature, and pH data. The existing standards and/or data sources included in the crosswalk for the hydrologic monitoring reporting format (Supplementary Table 1) were chosen for inclusion given their common use in the scientific community. They aligned generally on the types of hydrologic metadata to record (e.g., information about dates and times as well as information about data collection sites) but had different terminology across each of the resources^35^. The development of the reporting format included a review of additional data sources and standards beyond those listed in the crosswalk (Supplementary Table 1).

## Supplementary information

Supplementary Table 1

Supplementary Table 2

Hydrologic monitoring crosswalk

Hydrologic monitoring data dictionary

Water and soil chemistry crosswalk

Water and soil chemistry data dictionary

Soil respiration crosswalk

Soil respiration data dictionary

Leaf-level gas exchange crosswalk

Leaf-level gas exchange data dictionary

Amplicon abundance table crosswalk

Amplicon abundance table data dictionary

Location metadata crosswalk

Location metadata data dictionary

Sample ID crosswalk

Sample ID data dictionary

CSV guidelines crosswalk

CSV guidelines data dictionary

file-level metadata crosswalk

file-level metadata data dictionary

dataset metadata crosswalk

dataset metadata data dictionary

File level metadata

## Data Availability

Each data reporting format and all supporting documentation are hosted on our GitHub Community Space^36^ and archived in the ESS-DIVE data repository^25–35^. The supplementary information for this manuscript is also archived in ESS-DIVE^60–147^.

## Code availability

We have made code available which enables file-level metadata extraction^51^ for files that adhere to the reporting format.
